# Compositional Optimization of Sputtered WO_3_/MoO_3_ Films for High Coloration Efficiency

**DOI:** 10.3390/ma17051000

**Published:** 2024-02-22

**Authors:** Zoltán Lábadi, Dániel Takács, Zsolt Zolnai, Péter Petrik, Miklós Fried

**Affiliations:** 1 Institute of Technical Physics & Materials Science, Centre for Energy Research, Konkoly-Thege Rd. 29-33, H-1121 Budapest, Hungary; labadi.zoltan@ek.hun-ren.hu (Z.L.); tdani98elektro@gmail.com (D.T.); zolnai.zsolt@ek.hun-ren.hu (Z.Z.); petrik.peter@ek.hun-ren.hu (P.P.); 2 Institute of Microelectronics and Technology, Kando Kalman Faculty of Electrical Engineering, Óbuda University, H-1084 Budapest, Hungary; 3 Department of Electrical and Electronic Engineering, Institute of Physics, Faculty of Science and Technology, University of Debrecen, H-4032 Debrecen, Hungary

**Keywords:** reactive sputtering, combinatorial sputtering, electrochromic materials, tungsten–molybdenum oxide, coloration efficiency

## Abstract

Thin films of mixed MoO_3_ and WO_3_ were obtained using reactive magnetron sputtering onto ITO-covered glass, and the optimal composition was determined for the best electrochromic (EC) properties. A combinatorial material synthesis approach was applied throughout the deposition experiments, and the samples represented the full composition range of the binary MoO_3_/WO_3_ system. The electrochromic characteristics of the mixed oxide films were determined with simultaneous measurement of layer transmittance and applied electric current through the using organic propylene carbonate electrolyte cells in a conventional three-electrode configuration. Coloration efficiency data evaluated from the primary data plotted against the composition displayed a characteristic maximum at around 60% MoO_3_. Our combinatorial approach allows the localization of the maximum at 5% accuracy.

## 1. Introduction

Under applied DC bias, transition metal oxides can change their color. This reversible color change is known as electrochromism. Potential applications for electrochromic windows include use in sensors, displays, and automotive rear-view mirrors.

The solid-state electro-chromic device consists of the electrochromic (WO_3_, MoO_3_), charge storage and electrolyte layers (Ta_2_O_5_) sandwiched between transparent conducting electrodes (TCE). Electrochromic features like coloration efficiency (CE), cyclic durability and kinetics of coloration cycles of tungsten oxide strongly depend on its structure, morphology, and composition, and therefore on the deposition methods and growth parameters.

Electrochromic phenomena are defined as color change caused by applied electric current. There are many types [1] of electrochromic materials such as TiO_2_, CrO, Nb_2_O_5_, SnO_2_, NiO, IrO_2_, WO_3_, [2,3] and MoO_3_ [4,5]. Transition metal (tungsten and molybdenum) oxide films are the most common materials for this purpose.

The most extensively researched metal oxide is tungsten oxide; a significant number of studies have been written about its technology, morphology, and optical properties [2,3]. Due to its similar features to tungsten oxide, molybdenum trioxide films can efficiently be deposited using several different methods including chemical vapor deposition (CVD) as well as radio frequency (RF) and reactive sputtering.

Further physical processes like atmospheric pressure chemical vapor deposition (APCVD), dipping, the sol–gel method [3,6,7], and sintering [8] are also proven to be applicable for WO_3_ layer formation. Although a molybdenum-trioxide-based device has lower CE compared to a WO_3_-based one, its optical absorption peak can be found closer to the human eye sensitivity peak, and this makes this material very attractive for electrochromic in many applications [4,5].

Despite the fact that tungsten and molybdenum oxides are both among the most extensively studied materials for electrochromic devices, relatively little effort has been made to understand the properties of mixed oxides [8,9,10,11,12,13,14,15,16,17]. Most of these works investigated only one composition, even only in the composition range of doping effect, i.e., low % of molybdenum oxide in WO_3_.

Arvizu and coworkers [8] investigated electrochromic properties of mixed W–Mo oxides deposited using DC magnetron co-sputtering. Their samples covered the composition range between 0 and 30% Mo content at eight different points. Their focus of investigation was on the cyclic durability and spectral properties of the samples. They found that, during the first coloration cycle, the amount of inserted charge is larger than that extracted, and this phenomenon increases towards larger Mo contents. The authors assigned the change in the transferred electric charge to trapped and accumulated lithium ions and therefore increasingly difficult intercalation. Their films attained a permanent yellowish coloration.

Cyclic durability measurements showed that the maximum wavelength of the absorption band showed a blue shift with an increasing cycle number, as compared to pure WO_3_, and the resulting films were greyish in the colored state. The authors concluded that the mixed oxides can be used for “smart windows” to obtain a more neutral dark color [8].

Prameela and coworkers [9] investigated mixed materials, but their study was limited to four discrete compositions, i.e., (MoO_3_)_x_ − (WO_3_)_1−x_ for x = 0, 0.2, 0.4, 0.6, and 0.8. Their XRD spectra showed that the WO_3_ grain size was decreasing with increasing MoO_3_ content. Raman modes of both MoO_3_ and WO_3_ were observed for x = 0.4. With the further increase in MoO_3_ content, Raman modes corresponding to MoO_3_ alongside an increase in intensity were observed. From these Raman and IR studies, they observed that vibration modes in WO_3_ were shifted due to the addition of MoO_3_.

Faughnan and coworkers [10] investigated co-evaporated W–Mo amorphous oxides with 0, 5, 35, and 75% Mo content. Their results show that electrochromic optical absorption of the samples appears at higher energy than in pure oxide alone. The authors explained this phenomenon with the intervalency charge transfer. The highest absorption peak of mixed oxides was found at 2.15 eV (1.4 eV for WO_3_). Since this is closer to the sensitivity maximum of the human eye, this is considered advantageous for electrochromic display devices.

Madhavi and coworkers [11] prepared pure and Mo-doped WO_3_ films on an ITO-coated glass substrate at 200 °C using RF magnetron sputtering. According to their observations, pure WO_3_ films demonstrated a dense surface morphology while Mo-doped layers contained grain-like agglomerates uniformly distributed on the surface of the sample. The electrochromic properties of the films were characterized using cyclic voltammetry in 1 M Li_2_SO_4_ electrolyte solution. They found that the optical transmittance of the 1.2% (n/n) Mo-doped tungsten oxide films decreased from 85% to 75%, whereas CE improved to 42.5 cm^2^/C compared to 33.8 cm^2^/C in pure WO_3_.

Ivanova and coworkers [12] developed a low-temperature atmospheric CVD process to deposit mixed W–Mo oxide films. The Mo content of their mixed sample was 7% (although the authors admit difficulties in reproducibility). The electrochromic characterization of MoO_3_ and mixed Mo/W oxide films was made using cyclic voltammetry performed in a conventional potentiostatic three-electrode arrangement. Simultaneous current and transmittance measurements were made at different wavelengths over the spectral range of 400–800 nm during the voltammetry sweeps. They found their CVD process suitable for electrochromic devices and found enhanced CE in Mo-containing samples.

Dong and coworkers [13] used room temperature electron beam evaporation to deposit various W–Mo mixed oxide electrochromic films with Mo/W atom ratios of 3:2, 1:1, and 1:2. The 40 nm thin mixed films displayed good overall electrochromic performance, with a high average transmittance of 77.4%, fast color-switching time (τ_c_ = 2.7 s and τ_b_ = 4.1 s), and high CE (70 cm^2^/C). The authors attribute the improved EC performances to electron intervalence transition together with the fast charge–transfer and ion–diffusion dynamics.

Zhou at al. [14] investigated metal doping during a sulfate-assisted hydrothermal method and found it to be an effective method for construction of Mo-doped WO_3_ nanowire arrays. The optimized Mo-doped WO_3_ nanowire arrays displayed advanced electrochromic properties with a fast switching speed (3.2 s and 2.6 s for coloration and bleaching, respectively), significant optical modulation (56.7% at 750 nm, 83.0% at 1600 nm, and 48.5% at 10 μm), high CE (123.5 cm^2^/C), and outstanding cycling stability.

Jittiarporn et al. [15] deposited hybrid WO_3_–MoO_3_ thin films deposited onto conductive indium tin oxide (ITO) glass substrates using a sol–gel dip-coating technique. They investigated the effect of the annealing temperature (200–500 °C), concentration of MoO_3_ (0–10%), and Pluronic P123 triblock copolymer (0–20% *w*/*v*) used as a template. The authors found the highest CE together with the best cycling stability and shortest response time when the 5% MoO_3_–95% WO_3_ films were prepared without template and annealed at 200 °C—which represented an overall improvement with respect to a WO_3_ film.

Wu et al. [16] prepared MoO_3–y_/WO_3–x_ films based on MoO_3–y_ nanosheets, which show a dark blue color, which is aligned to the human eye sensitivity and meets visual comfort requirements compared to pure WO_3–x_ film. Their device, based on the MoO_3–y_/WO_3–x_ film, exhibited high optical modulation in the whole visible range and could operate at self-powered mode with a quick response and outstanding cycle stability. Moreover, they developed an ionic writing board based on a MoO_3–y_/WO_3–x_ mixed oxide system to overcome the fixed display information matter in conventional electrochromic displays.

Hsu et al. [17] prepared WO_3_ nanoparticles and MoO_3_ nanoribbons using a microwave-enhanced hydrothermal method. They obtained a stable WO_3_/MoO_3_ suspension with a nanoheterojunction structure after using a grinding treatment. Furthermore, excess oxygen vacancies were introduced in the MoO_3_ nanoribbons (MoO_x_) during the grinding process. The ground WO_3_/MoO_x_ composite film was found to have improved electrochromic (EC) performance compared to the pure ground WO_3_ and MoO_3_ ones. This was attributed partly to its favorable morphology for ion transport, and partly to the enhanced intervalence electron transfer in the mixed oxide.

Although the EC effect can be more pronounced in mixed oxides due to possible electron transitions between two sets of electron states, relatively few studies deal with the possible optimization of the EC parameters in mixed oxide type films.

In the frame of this article, we aim to study the electrochromic behavior of mixed tungsten and molybdenum oxides deposited using reactive DC magnetron sputtering. It should be noted that we apply a combinatorial approach for composition-graded layer deposition in order to allow study samples chosen from a full and continuous WO_3(x)_ − MoO_3(1−x)_ composition range. We are not aware of any publications that have used a combinatorial approach in this field. The deposited films were characterized using spectroscopic ellipsometry (SE), Rutherford backscattering spectrometry (RBS), and CE measurements.

## 2. Materials and Methods

### 2.1. Description of the Sputtering Facility

Layer deposition experiments were carried out in a pulsed-mode DC reactive magnetron sputtering facility equipped with Mo and W metallic targets. High-purity (99.95%) targets with a size of 110 mm × 440 mm were used. Depositions were made onto a 30 cm × 30 cm soda–lime glass (SLG) and/or any smaller size Si wafer or ITO-covered glass substrate. An Ar + O_2_ atmosphere composition was set in 5% mass flow controller steps. A uniaxial substrate transport system kept the substrate in constant linear motion at a typical speed of 25 cm/s.

Table 1 shows the relevant typical parameters for the combinatorial deposition.

### 2.2. Combinatorial Deposition

**Figure 1:**
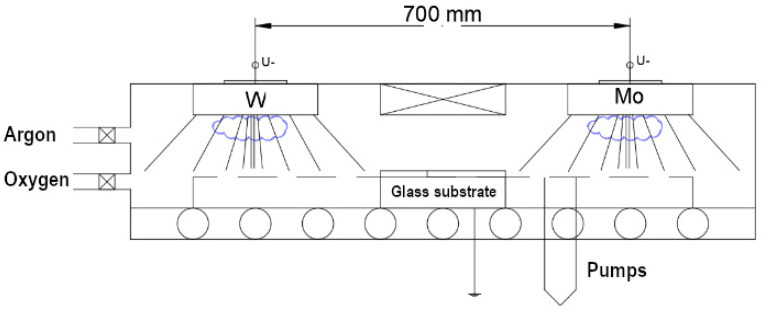
Cross-sectional schematic view of the sputtering chamber and the target arrangement.

Figure 1 shows the schematic cross-section of the sputtering chamber. The arrangement of the targets provides a non-uniform flux of sputtered material along the horizontal axis. Since the substrate performs a uniaxial forward and back movement, proper choice of the left and right movement limits can result in a horizontal composition gradient on the substrate.

An observer travelling on the left edge of the substrate experiences WO_3_ flux only. Similarly, pure MoO_3_ deposition occurs on the right edge. Furthermore, a continuous composition gradient is observable between the two sides.

Actual layer depositions were carried out in a reactive (Ar + O_2_) gas mixture in high vacuum (~2 × 10^−6^ mbar base and ~10^−3^ mbar process pressure). A 30 sccm/s Ar and 30 sccm/s O_2_ volumetric flow rate was applied in the magnetron sputtering chamber. The substrates were 30 × 30 cm^2^ soda–lime glasses. The whole process was accomplished in constant power control mode for P = 1000 W for the W target and P = 1250 W for Mo, respectively. A 25 cm/s walking speed (back and forth between the edges) was chosen in order to ensure the intermixing in between the individual sublayers (i.e., to avoid superlattice formation). In total, 1000 cycles were needed to deposit a layer of approximately 300 nm in the middle range of the substrate glass.

**Figure 2:**
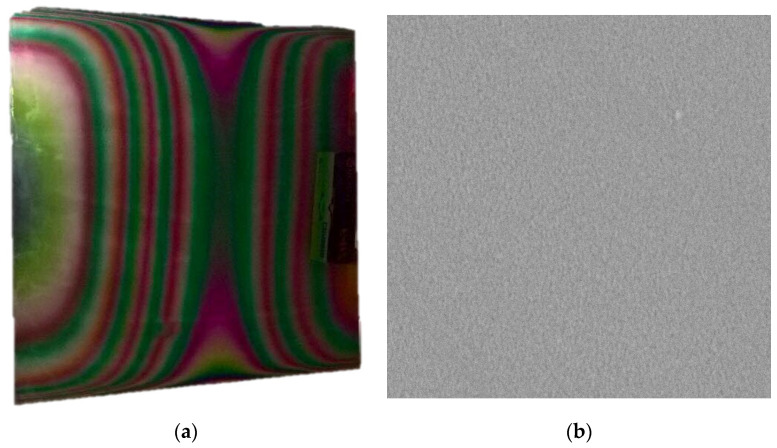
(**a**) Photo of 30 × 30 cm^2^ substrate with composition gradient layers. Interference bands show thickness and composition gradient; (**b**) 4 × 4-micron SEM micrograph from the center part of the 10 cm-long Si stripe on the upper part of Figure 3a.

Figure 2 shows the final lateral structure of the layer with interference bands, which clearly indicate the thickness and composition inhomogeneity. A W-layer was applied in this case to make a contrast between the mixed metal-oxide layer and the glass substrate.

**Figure 3:**
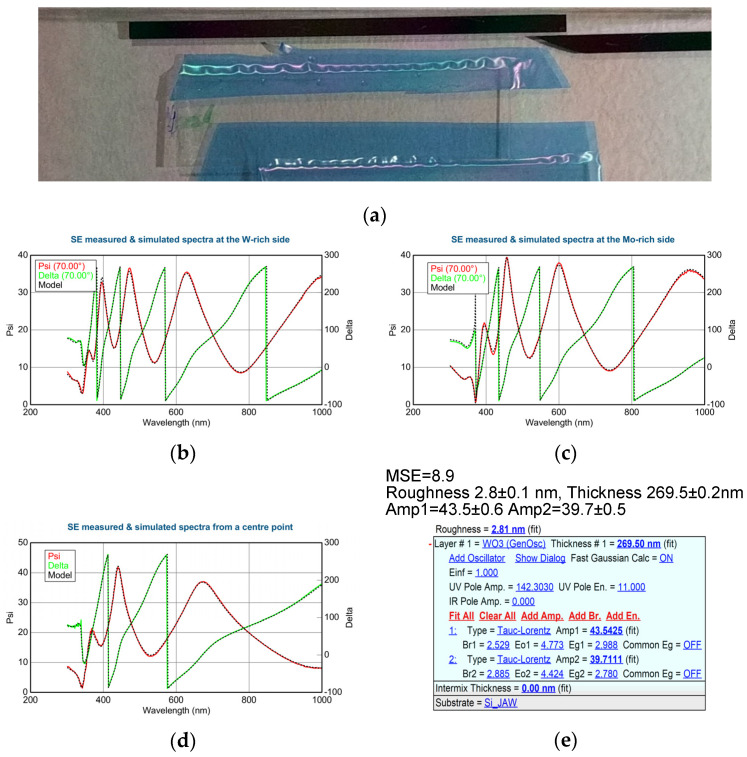

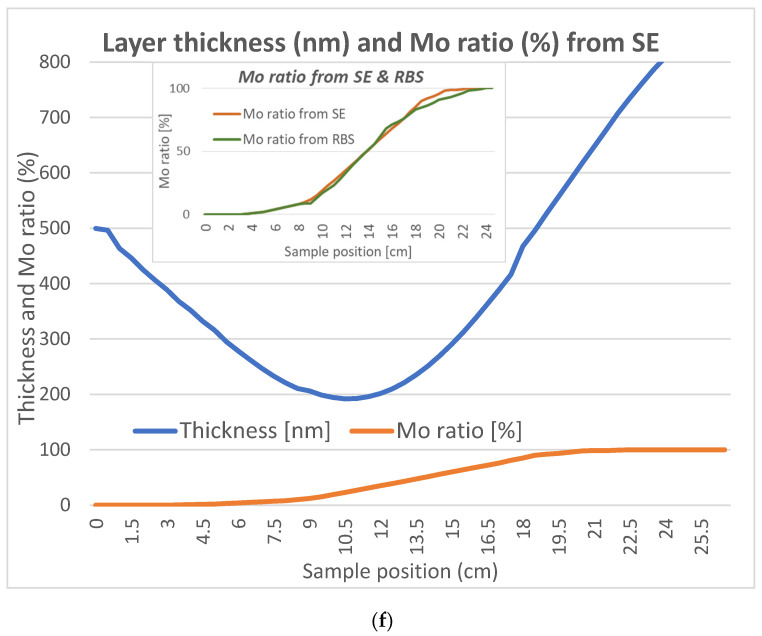
(**a**) Layout of the WO_3_/MoO_3_/ITO-covered glass and Si samples (note the blue tape masking the ITO layer for contacts); (**b**) measured and fitted SE curves of WO_3_; (**c**) combinatorial WO_3_/MoO_3_ (**d**) MoO_3_ layer; (**e**) with the T–L oscillator optical model and the values of the fitted parameters with errors; (**f**) layer thickness and Mo ratio vs. sample position. Insert shows the RBS and SE together.

To facilitate characterization, silicon stripes were placed onto the substrate glass during the sample preparation; see Figure 3a. Furthermore, a set of 25 mm-by-75 mm microscope slide glasses covered by a 60 nm-thick ITO layer was also arranged on the main glass. A 5 mm-by-75 mm area of these slides was covered with masking tape in order to provide a contact area for the electrochemical half-cell. These smaller pieces served as transparent electrodes for the electrochromic characterization. All photographs were made with a normal digital camera: 12 MP Sony sensor, F1.8 OIS Dual Pixel AF.

### 2.3. Electrochemical Measurements

To measure the CE as a function of binary composition, the glass samples covered by ITO and mixed oxide were positioned into a purpose-made electrochemical cell between two parallel windows.

The cell was filled with 1 M lithium perchlorate (LiClO_4_)/propylene carbonate electrolyte. The masked area of the slides remained above the liquid level, allowing electric contact onto the ITO layer. Finally, a Pt wire counter electrode was installed into the electrolyte alongside a reference electrode. This arrangement provided the fully functional electrochromic cell.

During coloration/bleaching, the following reversible electrochemical reaction takes place:

Mo_x_W_1−x_O_3_ + yLi^+^ + ye^−^ → Li_y_Mo_x_W_1−x_O_3_ (1)

Controlled current was applied through the cell using a Farnell U2722 Source Measurement Unit (SMU) to register the current through coloration and bleaching cycles of the electrochromic layer. Simultaneous spectral transmission measurements were made by setting the Woollam M2000 ellipsometer to transmission mode.

A Woollam M2000 SE device was applied to map the thickness and composition change. Woollam Co. CompleteEASE v. 5.15 software was used to evaluate the data with built-in optical models and oscillator functions: Tauc–Lorentz (T–L) oscillators or effective medium approximation. We checked the SE results using RBS. The method and background are described in a separate article [18].

Mapping measurements were performed with a mm-sized beam spot with one spectrum pair per 5 mm.

## 3. Results and Discussion

### 3.1. Characterization of the WO_3_/MoO_3_ Samples Using SE and RBS

Following the sputtering process, the samples were investigated and mapped using SE, which is a fast, cost-effective, and non-destructive contactless method. SE measurements were carried out on 5 mm-wide silicon probes covering the whole width of the substrate glass. Since the chamber mechanism enables only uniaxial movements, any gradient along the perpendicular axis was ruled out.

Taking into account the total thickness range of the film (see Table 2) and the applied number of cycles, the superlattice formation (i.e., formation of individual sublayers) was ruled out. So, the material can be considered as an atomically mixed alloy. We determined the dispersions for pure (100%) WO_3_ and MoO_3_ using the T–L oscillator model; see the inserted optical model in Figure 3e. We used the determined complex refractive indices (and complex dielectric functions) of the pure WO_3_ and MoO_3_ using the T–L oscillator model. Finding the best match (see the SE spectra in Figure 3b–d) between the model and the experiment was systematically achieved through finding regression minima. The mean squared error (MSE) value, which is lower than 10, is very good in SE. We used five fitted parameters (Figure 3e bolded parameters) at each sample point: the layer thicknesses and the two amplitudes (oscillator strengths). Three basic parameters (i.e., broadening, peak position, and bandgap energy) of the pure materials were determined from the measurements made near the edges of the samples; see Figure 3b “W-rich” and Figure 3d “Mo-rich”. The Mo ratio was determined from the fitted SE parameters: ratio of the Amp1 and Amp2 parameters relative to the 100% WO_3_ and MoO_3_ data.

**Figure 4:**
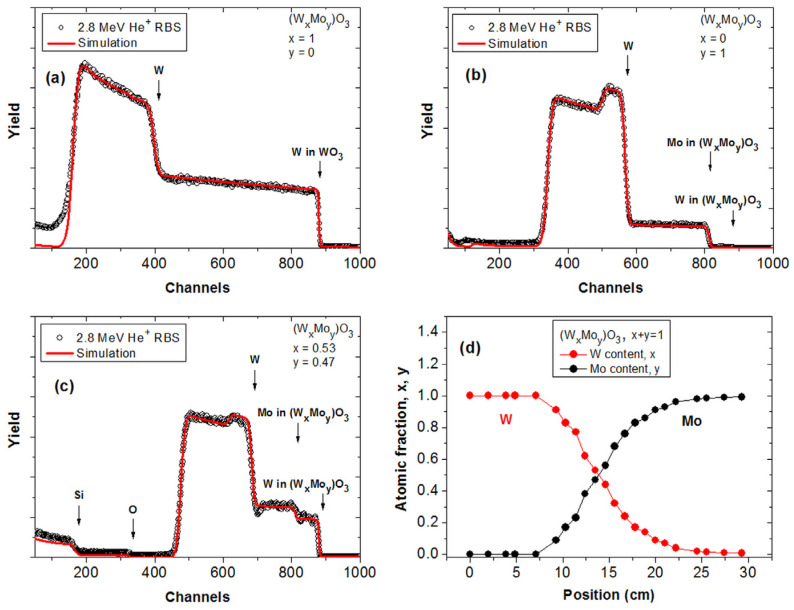
Measured and simulated (fitted) RBS spectra from the W-rich side (**a**), from the Mo-rich side, (**b**) and from near the center position (**c**) of the sample, together with a composition map along a line as evaluated from RBS data (**d**).

In order to prove the composition data determined from the SE data (the ratio of the Amp1 and Amp2 parameters relative to the 100% WO_3_ and MoO_3_ data), RBS was applied as an independent characterization method. We used the same analysis, which was used and explained in our previous paper [18]. Figure 4 shows RBS spectra measured using a 2.8-MeV energy He^+^-beam at a backscattering angle of 165° and sample tilt angle of 7°. The spectral edge for W, Mo, and O in the (W_x_Mo_y_)O_3_ layer and for Si in the substrate are indicated. The thickness and atomic composition for a given layer in the sample have been evaluated from the measured spectrum width and height, respectively, using the RBX simulation code [19]. The RBS spectra in Figure 4a–c represent typical examples for a W-rich region, a Mo-rich region, and the central region of the sample with almost equal Mo and W content. Variation in the (W_x_Mo_y_)O_3_ composition is reflected in the different Mo-to-W RBS yields. In Figure 4d, W and Mo atomic fractions in the (W_x_Mo_y_)O_3_ layer, evaluated from RBS, can be seen as a function of position measured along the central line of the sample. Table 2 shows the results of the RBS measurements compared with the SE results. We found good agreement within a 2% error. This error originated partly from the two methods and the position ambiguity (originated from the mm-size beam spot of SE device) during the two independent measurements. The ellipsometry mapping (Figure 3) and RBS data (Figure 4) provide the necessary thickness and composition data, which, together with the transmission and current measurements, allow the calculation of the coloration efficiencies. This means a 2% error in the determined optimal composition.

### 3.2. Coloration Efficiency Measurements

CE is defined as the change in optical density (ΔOD) per unit of charge of insertion or extraction and is given using the following relation:

CE = ΔOD Δ*Q* = log (*T_a_*/*T_b_*) Δ*Q*, (2)

where ΔOD is the change in optical density, Δ*Q* is the charge density (C/cm^2^), and *T_b_* and *T_c_* are the optical transmittances in the bleached and colored states, respectively. Transmittances were directly measured during the coloration process, while the charge was calculated from the integral of the current vs. time data and the electrolyte wetted area of the sample.

**Figure 5:**
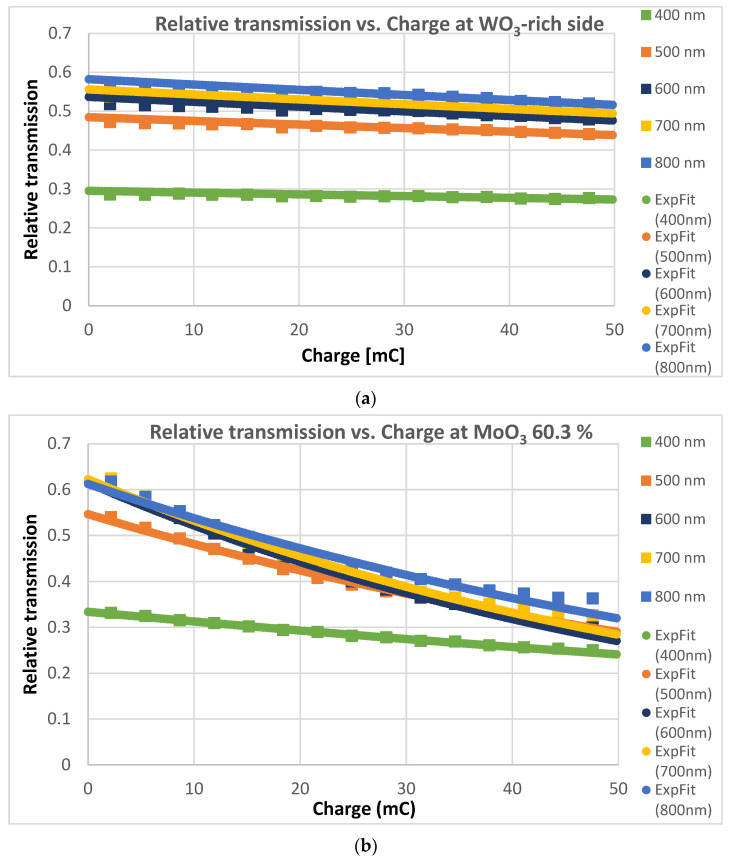

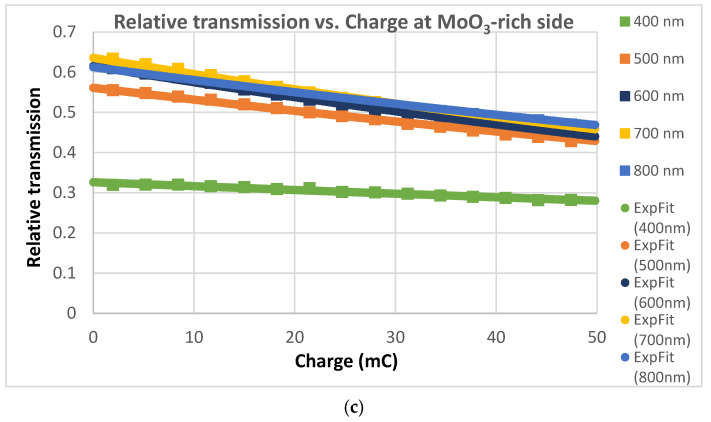
Relative transmission vs. input charge curves at five wavelengths at the W-rich side (**a**), at the Mo-60.3% (**b**), and at the Mo-rich side (**c**). CE values were determined from the fitted exponential curves (ExpFit).

CE values were determined at each sample position from the relative transmission vs. input charge curves. Three examples are presented in Figure 5, one at the W-rich side, one at the Mo-60.3%, and one at the Mo-rich side. We show the relative transmission curves at five wavelengths (400–800 nm visible range) at each position and the fitted exponential curves.

**Figure 6:**
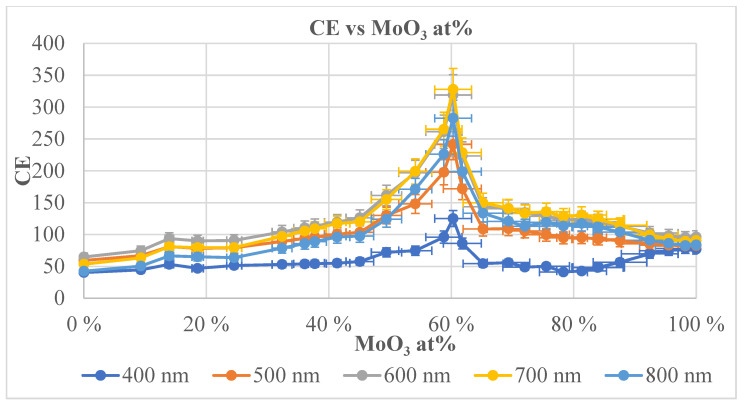
Calculated CE data as a function of MoO_3_ fraction of the layer including the proper error bars (individual color-coded curves represent different wavelengths).

**Figure 7:**
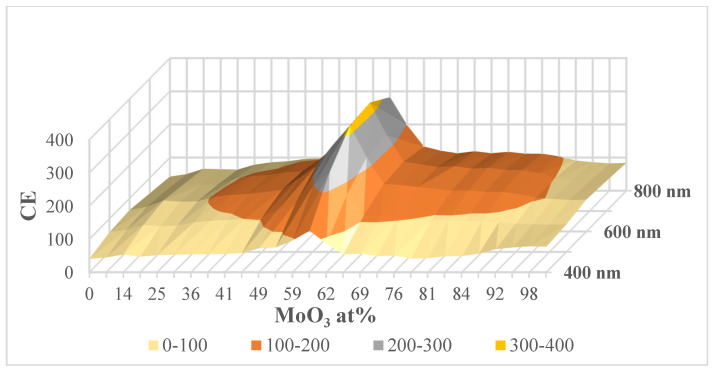
Calculated CE data as a function of MoO_3_ fraction—3D graphical representation.

Figure 6 shows the calculated CE data as a function of MoO_3_ fraction of the layer (individual color-coded curves represent different wavelengths, while Figure 7 is a 3D representation of the data. Individual points were calculated from the average of three independent measurements. Horizontal error bars are calculated on the basis of the accuracy of sample positioning in the measuring cell and the spot size of the optical beam.

The CE measurements (summarized in Figure 6) clearly show that a characteristic maximum of CE exists at: the 60% Mo fraction. Furthermore, this maximum is present throughout the 400–800 nm visible spectral range, and its relative intensity increases towards the red end of the spectrum. The spectral maximum is in the 600–700 nm wavelength region, and the CE decreases at 800 nm and in the near-infrared region.

A possible explanation of the positive anomaly of the CE values can be related to the distribution of non-stoichiometric excess oxygen between the W and Mo sub-phases. Inamdar and coworkers [20] studied coloration efficiencies of sputtered WO_3+δ_ films and found that CE forms a peak near δ~0.3 in the oxygen over-stoichiometric region (δ > 0). It should be noted that they observed a relatively sharp maximum and at least twofold increase in CE. They concluded that δ~0.3 is the optimum value, which offers a larger density of Li^+^ ion intercalation/deintercalation, resulting in the highest CE value.

Based on this result, the maximum in the CE as a function of increasing Mo content can be interpreted as follows: as the W content of the layer decreases, the constant oxygen partial pressure results in a relatively increasing δ value, which in turn increases the CE of the film. However, this effect is accompanied by the simultaneous decrease in the W content, thus forming a maximum-type dependence of CE as a function of composition.

Evaluation of the previous data of electrochromic properties measured at different but always discrete compositions [8,9,10,11,12,13,14,15,16,17] led to the general conclusion that energy of the maximum of the absorption band of the mixed oxide films shifts towards higher energy as compared to pure WO_3_. Our experiences gained throughout the CE measurements cycles are in agreement with the results of Ref. [8]. The resulting films exhibit a greyish color in the colored state. Hence, the mixed oxides can be used for “smart windows” in order to obtain a more neutral dark color. However, the existence of a characteristic maximum of CE in the mixed oxide composition range offers a further argument for the use of WO_3(0.4)_MoO_3(0.6)_ mixed oxide for enhanced performance electrochromic films.

## 4. Conclusions

In this study, we present a method for combinatorial deposition of WO_3_/MoO_3_ mixed oxides using reactive magnetron sputtering. Deposition covered the whole composition range of this binary system, and samples were characterized using SE and RBS. The composition gradient enabled the testing of electrochromic properties at 5% composition steps. A sharp maximum in CE was observed at 60% Mo content. The determined optimal composition has a 2% error. This error originated partly from the two methods and the position ambiguity (originated from the mm-size beam spot of SE device) during the two independent measurements.

Electrochromic device developers can use these results to optimize the CE by finding proper thickness and after-deposition (annealing) treatment using this optimal composition.

## Figures and Tables

**Table 1:** Typical sputtering parameters for the combinatorial deposition.

Deposition Parameter	Typical Value
Working distance (mm)	60
Substrate preheating	Optional, up to 300 °C
End vacuum (mbar)	1–5 × 10^−6^
Target power (W)	500–4000
Target voltage (V)	280–380
Pulse duty factor (µs)	0.5
Pulse cycle time (µs)	10

**Table 2:** Layer thickness and Mo ratio results from SE and RBS analysis.

Position (cm)	Thickness (nm)	Mo % from SE	Mo % from RBS
0	499.5	0	-
0.5	496.4	0	-
1	463.3	0	-
1.5	446.1	0	-
2	424.5	0	0
2.5	406.1	0	-
3	388.8	0	-
3.5	367.7	0.5	-
4	351.7	1	0
4.5	332.1	1.5	-
5	316.1	2	-
5.5	295.0	3	-
6	278.4	4	-
6.5	262.3	5	-
7	247.0	6	6
7.5	232.9	7	-
8	220.8	8	-
8.5	210.7	10	-
9	206.2	12	-
9.5	198.9	15	-
10	194.3	19	17
10.5	192.0	23	-
11	192.5	27	-
11.5	195.9	31	23
12	201.6	35	-
12.5	210.0	39	38
13	221.2	43	-
13.5	235.2	47.5	47
14	251.4	51.	-
14.5	270.0	56	-
15	290.6	60	-
15.5	313.0	64	68
16	337.2	68	-
16.5	362.8	72	76
17	389.2	76	-
17.5	416.6	81	-
18	467.4	85	83
18.5	495.4	90	-
19	526.8	92	86
19.5	556.8	93.5	-
20	586.9	95.5	91
20.5	617.5	98	-
21	647.1	98.5	93
21.5	676.9	98.5	-
22	707.4	99	96
22.5	734.6	100	98
23	760.7	100	-
23.5	786.0	100	-
24	808.8	100	-
24.5	831.2	100	-
25	852.1	100	-
25.5	873.2	100	100
26	889.2	100	100

## Data Availability

Data are contained within the article.
